# Community-based integrated medical and elderly care in Chengdu, China: organisational heterogeneity, service-capacity and service-receipt patterns, and implications for care continuity

**DOI:** 10.3389/frhs.2026.1875860

**Published:** 2026-10-19

**Authors:** Xianmin Wang, Yuxuan Peng, Jing Liu, Ming Bai, Jiawei Peng, Chi Heng, Yichen Yang, Hui Lu, Xin Wang, Chunrong Li, Tongyong Luo

**Affiliations:** 1 Sichuan Provincial Women’s and Children’s Hospital/Affiliated Women’s and Children’s Hospital of Chengdu Medical College, Chengdu, China; 2 Chengdu Eighth People’s Hospital/Geriatric Hospital of Chengdu Medical College, Chengdu, China; 3 School of Public Health, Chengdu Medical College, Chengdu, China; 4 Shanghai University of International Business and Economics, Shanghai, China

**Keywords:** China, community-based care, health policy, integrated care, long-term care, older adults

## Abstract

**Background:**

Community-based integrated medical and elderly care (IMEC) has expanded in China, but its organisational capacity, information-related service functions and acceptability among older adults remain insufficiently understood. These issues are relevant to care continuity because integrated care depends not only on organisational arrangements but also on the ability to maintain usable health information and coordinate services across care settings.

**Methods:**

In a cross-sectional study conducted in Chengdu between October 2023 and May 2024, we analysed two parallel surveys: an institutional survey that initially covered 132 registered IMEC institutions, of which 127 were included in the final analysis, and a household survey of 2,525 adults aged ≥65 years recruited from selected communities. We presented institutional service-capacity indicators and residents’ reported service receipt and perceived importance in parallel across selected care domains and used multivariable logistic regression to examine factors associated with willingness to choose community-based IMEC.

**Results:**

Size-standardised analyses showed that medical-run care institutions had higher medical-bed proportions and medical staffing densities per 100 beds, whereas care-run medical institutions had a higher proportion of long-term-care beds. Elderly-care attendant density per resident did not differ significantly between the two models. Overall service-portfolio breadth was similar between the two organisational models. Overall, 68.8% of residents reported willingness to choose community-based IMEC. Higher willingness was associated with greater perceived need for convenient home/community medical services and awareness of community-based integrated care. Parallel descriptive analyses showed substantial perceived-need–capacity differences within institutions and importance–receipt differences among residents, particularly for home-based medical services, rehabilitation, psychosocial support and related community-oriented functions.

**Conclusions:**

Community-based IMEC in Chengdu showed substantial organisational heterogeneity, while the two surveys identified distinct service-capacity and service-receipt patterns relevant to ageing in place and care continuity. Strengthening community-oriented service packages, service visibility and information-related capacities may better support ageing in place and future data-supported continuity of care.

## 1 Introduction

Population ageing is transforming health and social care systems worldwide. Increased longevity has been accompanied by a rising prevalence of multimorbidity, chronic disease, functional decline and long-term support needs, exposing the limitations of systems that remain largely organised around acute and episodic treatment (1–3). In response, integrated care has become a central policy and service strategy for connecting medical care, long-term care and social support, particularly for older adults whose needs extend beyond disease-specific treatment alone (4–7).

Within this agenda, ageing in place has emerged as a widely endorsed objective. It reflects both older adults’ preference to remain in familiar homes and communities and policy concerns about the costs and sustainability of institution-centred care (8–10). However, ageing in place is not achieved simply by relocating care from institutions to communities. It requires accessible primary and community medical care, rehabilitation, social support, health education, psychosocial care and effective coordination across sectors (11–13). International evidence suggests that integrated care reforms can improve coordination and user experience in some settings, but their effects are often mixed and context-dependent, especially where financing, workforce capacity and local service organisation remain fragmented (14–17). Continuity of care in such settings increasingly depends on the availability and flow of personal health information across care providers and service settings. Personal health data spaces (PHDSs) and related data-sharing infrastructures have been proposed as ways to enable individuals’ health information to follow them across providers, time points and care contexts, while supporting appropriate access, coordination and privacy protection (18–20). For older adults receiving integrated medical and elderly care, this means that organisational integration alone is insufficient: continuity also requires basic information-related capacities, such as establishing health records, maintaining usable care information and supporting data-informed coordination between home-, community-, outpatient- and institution-based services.

China provides a particularly important setting in which to examine these issues. Rapid population ageing has coincided with declining family size, increased migration and changing intergenerational care arrangements, weakening the capacity of traditional family-based elder support and increasing reliance on formal health and care services (21–24). Since the mid-2010s, national policy has strongly promoted the integration of medical and elderly care services as part of broader health and long-term care reform (25, 26). Evidence from China indicates that implementation remains uneven, with persistent challenges in governance, workforce capacity and local service organisation (22, 27). Financing arrangements, including the evolving role of long-term care insurance, may further shape healthcare utilisation and formal care provision among older adults (28–30).

Within this policy framework, integrated medical and elderly care (IMEC) institutions have become a key organisational vehicle for putting integration into practice. In this study, community-based IMEC refers to institution-based service organisations intended to support older adults living in the community through combined medical, nursing, rehabilitation and related care services, rather than to a single uniform model of community care. Yet IMEC in China is not a single standardised model. In practice, IMEC institutions commonly develop through two organisational pathways. Some institutions have developed from medical providers that expanded into long-term care (“medical-run care”), whereas others originated as elderly care facilities that later added medical services (“care-run medical”). That is, “medical-run care” institutions are medical providers that extend into long-term care, whereas “care-run medical” institutions are older-adult care providers that subsequently add medical functions. These different institutional pathways reflect the historical separation between the health and social care sectors and are likely to shape staffing structures, bed composition, service portfolios, charging patterns and degrees of community embeddedness (21, 27, 31). This heterogeneity is especially relevant in large urban regions where ageing pressures, service infrastructure and geographical accessibility differ markedly across districts. Chengdu is a useful case in this respect: the proportion of adults aged 65 years and older varies substantially across its inner-, middle- and outer-ring districts, from 9.71% in Pidu District to 21.48% in Jianyang, indicating that the demand for community-based integrated care is likely to be spatially uneven.

Despite increasing policy attention, two important gaps remain in the literature. First, much of the existing Chinese evidence has focused on policy development, system reform or institutional characteristics, with less attention to how integrated care is perceived and valued by community-dwelling older adults themselves (27, 31). Second, relatively few studies have linked residents’ willingness to choose integrated care with the actual service configurations of local IMEC institutions. This omission matters because willingness is unlikely to be an abstract attitude alone. Rather, it may reflect concrete expectations regarding medical access, rehabilitation, cost, continuity, home-based support and the perceived usefulness of available services. Recent Chinese studies suggest that utilisation or acceptance of home- and community-based medical and elderly care services is shaped not only by health needs but also by service accessibility, user awareness, affordability and local service conditions (32–34). Consistent with this, discrete choice evidence indicates that older and middle-aged adults in China make explicit trade-offs between service attributes such as medical capacity, quality and price when evaluating IMEC institutions (35). Whether current institutional service portfolios align with these priorities, however, remains insufficiently understood.

Against this background, this study examines community-based integrated medical and elderly care in Chengdu, China, from institutional and resident perspectives. By combining an institutional survey of 127 IMEC facilities with a household survey of 2,525 older adults recruited from selected communities, we address three questions. First, how do the two organisational models differ in bed composition, size-standardised staffing characteristics and service portfolios? Second, what service-capacity patterns are reported by institutions and what service-receipt and perceived-importance patterns are reported by older adults across selected care domains, including the information-related function of health record establishment? Third, which factors are associated with older adults’ willingness to choose a community-based IMEC model? Because the institutional and resident surveys were not directly connected at the district, community or facility-catchment level, the institutional–resident aggregate comparison in this study is descriptive and aggregate rather than relational or causal. By bringing organisational heterogeneity, institutional service-capacity patterns, resident service experiences, and user willingness and the basic information-related function of health record establishment into a single analytical framework, this study aims to clarify service-system conditions relevant to community-based IMEC, ageing in place and future data-supported continuity of care.

## 2 Materials and methods

### 2.1 Study design and setting

This cross-sectional study was conducted in Chengdu, a major city in south-west China, to examine community-based integrated medical and elderly care (IMEC) from both the supply and demand sides. Two parallel cross-sectional surveys were undertaken between October 2023 and May 2024: an institutional survey of IMEC facilities and a household survey of older adults recruited from selected communities. By combining these datasets, the study aimed to assess organisational heterogeneity across IMEC institutions, describe institutional service provision and older adults’ service experiences and priorities in parallel, and identify factors associated with willingness to choose a community-based IMEC model. Because the institutional and household surveys were not directly connected at the district, community, or facility-catchment level, the institutional and resident findings are presented in parallel for descriptive purposes rather than as a direct relational or supply–demand analysis.

Chengdu is a rapidly ageing large city that has actively promoted integrated medical and elderly care. To improve geographic coverage and capture variation in local care needs and service environments, study sites were organised across Chengdu's inner-, middle- and outer-ring districts/counties.

### 2.2 Institutional survey

The institutional survey was designed as a roster-based survey of registered IMEC providers in Chengdu rather than as a sampled facility survey. All 132 institutions that were formally registered and listed in the local administrative roster of IMEC providers during the study period were approached. Of these, 127 returned questionnaires with sufficient information for analysis and were included in the analytic sample; five did not return questionnaires with sufficient information for analysis and were excluded.

A structured facility questionnaire was administered by trained investigators between October 2023 and May 2024. The questionnaire collected data on:

1. organisational model;
2. bed capacity, including total beds, medical beds and elderly care beds;
3. resident composition by functional status, including numbers of self-care, partially dependent and dependent residents;
4. staffing structure, including medical doctors, medical nurses, medical technicians, medical care assistants, elderly-care doctors, elderly-care nurses, elderly-care technicians and elderly-care attendants;
5. monthly care fees by dependency level; and
6. service provision across key IMEC domains.

For each service domain, institutions reported whether they considered the service to be needed by community-dwelling older adults and whether they were currently able to provide the corresponding community-based service. Responses were coded dichotomously as yes or no. Operational definitions and coding of the key service-related measures are provided in Supplementary Table S1.

### 2.3 Household survey

The household survey included 2,525 adults aged 65 years or above recruited from selected communities. The resident sample was obtained using a geographically distributed convenience-based recruitment strategy. Communities were selected pragmatically across Chengdu's inner-, middle- and outer-ring areas to achieve broad geographic coverage across areas with differing age structures and service environments rather than through random selection from all communities in Chengdu. Within selected communities, eligible older adults were recruited during community-based fieldwork by trained interviewers, with assistance from local community workers where appropriate. No hypothesis-specific *a priori* power calculation was used for the household survey; the achieved sample size was determined pragmatically by fieldwork scope and feasibility, with the aim of obtaining broad geographic coverage across Chengdu's inner-, middle- and outer-ring areas.

The household sample should therefore be interpreted as a geographically distributed convenience sample rather than a random, quota-based or population-weighted probability sample of all older adults in Chengdu. Sampling weights were not applied. Community-level identifiers and participant counts by individual community were not retained in the final analytic dataset; therefore, the exact number of participating communities and the distribution of participants across individual communities could not be reliably reconstructed for the present analysis. Counts of older adults approached, refusals, non-response and non-participation were likewise not retained. The achieved sample included 28 respondents who reported institutional care within the community as their current main care arrangement. We therefore describe the sample as older adults recruited from selected communities rather than as exclusively community-dwelling older adults.

### 2.4 Measures

#### 2.4.1 Outcome variable

The primary outcome was willingness to choose a community home-based IMEC model. This variable was measured using a single questionnaire item translated from Chinese as: “Would you be willing to choose a community home-based integrated medical and elderly care model?” Response options were “yes” and “no”, and the variable was coded dichotomously as willing vs. unwilling. This item captured broad stated willingness toward the model rather than willingness to use any specific service modality, such as home-based services, community day care, outpatient services or residential institutional care. In the current sample, 1,738 respondents (68.8%) were classified as willing and 787 (31.2%) as unwilling.

#### 2.4.2 Institutional characteristics

Institutional characteristics used to compare organisational models included total beds, medical beds, elderly care beds, numbers of residents by dependency level, monthly fees, and staffing composition. Organisational type was classified as medical-run care institution (medical providers that have expanded into elderly care) or care-run medical institution (elderly-care providers that have added medical service capacity). These variables were selected to characterise structural and functional heterogeneity across institutions operating under the IMEC label.

To account for differences in institutional size, additional size-standardised indicators were calculated at the facility level. These included medical beds and long-term-care beds as percentages of total beds; medical doctors, nurses, technicians and their combined total per 100 beds; and elderly-care attendants per 100 residents and per 100 beds. Ratios were calculated for each institution before group-level comparison. Institutions with a zero denominator were excluded only from the corresponding ratio analysis. Service-portfolio breadth was defined as the number of 12 prespecified service domains that each institution considered to be needed by community-dwelling older adults and the number that it was currently able to provide.

#### 2.4.3 Resident characteristics

Resident-level variables included sex, living arrangement, main caregiver for daily life, number of chronic conditions, ADL status, disability level, monthly out-of-pocket medical expenditure, acceptable monthly payment for integrated services, preferred payment method, signed family doctor/community team contract, awareness of community-based integrated care, current main care arrangement and perceived need for convenient home/community medical services. Exact age, education, household income and district/ring of residence were not collected in the household questionnaire and were therefore unavailable for the present analyses. All respondents met the eligibility criterion of being aged 65 years or older.

For regression analysis, chronic disease burden was grouped as none, 1–2 conditions, and ≥3 conditions; ADL status was grouped as fully independent, partially dependent and fully dependent; and perceived need for convenient home/community medical services was collapsed into high perceived need vs. lower perceived need, consistent with the final multivariable model reported in Table 1. The exposure variables ultimately retained in the adjusted model were female sex, chronic disease burden, ADL status, monthly out-of-pocket medical expenditure, signed family doctor/community team contract, awareness of community-based integrated care and high perceived need for convenient home/community medical services.

**Table 1:** Multivariable logistic regression of factors associated with willingness to choose a community home-based IMEC model.

Variable	aOR	95% CI	*P*-value
High perceived need for convenient home/community medical services	2.76	2.23–3.42	<0.001
Female (vs. male)	1.10	0.92–1.31	0.280
1–2 chronic conditions (vs. none)	1.03	0.84–1.25	0.796
≥3 chronic conditions (vs. none)	0.98	0.66–1.45	0.928
Partially dependent (vs. fully independent	0.78	0.59–1.03	0.085
Fully dependent in ADL (vs. fully independent)	1.34	0.82–2.18	0.247
OOP 100–499 RMB (vs. <100)	1.15	0.93–1.43	0.182
OOP 500–999 RMB (vs. <100)	1.38	1.01–1.87	0.040
OOP 1,000–1,999 RMB (vs. <100)	1.14	0.77–1.70	0.511
OOP 2,000–2,999 RMB (vs. <100)	1.29	0.66–2.50	0.454
OOP ≥3,000 RMB (vs. <100)	1.00	0.46–2.16	0.996
Signed family doctor/community contract (vs. no)	1.01	0.81–1.26	0.895
Aware of community-based integrated care (vs. not aware)	1.55	1.28–1.87	<0.001

#### 2.4.4 Parallel descriptive presentation of service-domain indicators and information-related care-continuity measure

To describe service-related patterns from the institutional and resident perspectives, selected service domains were presented in parallel across the two surveys. On the institutional side, each domain was represented by whether institutions considered the service to be needed by community-dwelling older adults and whether they were currently able to provide the corresponding service. On the resident side, corresponding indicators captured whether respondents had ever received the service and whether they considered it important.

These indicators were not treated as directly equivalent measures. In particular, resident-reported service receipt was not linked to the surveyed IMEC institutions, and the source of previously received services was not recorded. The institutional and resident indicators were therefore juxtaposed for descriptive purposes only. Within the institutional survey, differences between “considered needed by community-dwelling older adults” and “currently able to provide” were described as perceived-need–capacity differences. Within the household survey, differences between “considered important” and “ever received” were described as importance–receipt differences. No direct supply–demand mismatch was inferred from the cross-dataset presentation.

Health record establishment was one of the institutional service domains included in the questionnaire. For this domain, institutions reported whether they considered the service to be needed by community-dwelling older adults and whether they were currently able to provide it. The item did not distinguish paper-based from electronic records and did not assess record updating, individual access or control, interoperability, consent or privacy mechanisms, or cross-provider data exchange. It was therefore treated only as a basic information-recording service indicator potentially relevant to continuity of care.

### 2.5 Statistical analysis

Descriptive statistics were used to summarise institutional and resident characteristics. For the institutional survey, most continuous indicators were non-normally distributed and were therefore presented as medians with interquartile ranges. Differences between medical-run care institutions and care-run medical institutions were tested using the Mann–Whitney *U* test. Categorical variables were summarised using frequencies and percentages. This strategy corresponds to the structure of Tables 2a, 2b.

**Table 2a:** Institutional beds, residents, and staffing by institutional model.

Variable	Medical-run care		Care-run medical		*U* statistic	*P* value
	P50 (P25, P75)	Min–Max	P50 (P25, P75)	Min–Max		
Total beds	150 (84, 207)	10–1,449	206 (110, 430)	12–1,707	1,489	0.032
Medical beds	90 (50, 146)	0–1,372	22 (0, 60)	0–400	2,902	<0.001
Long-term care beds	32 (30, 80)	0–532	155 (87, 304)	5–1,681	766	<0.001
Number of residents	30 (8, 72)	0–1,374	122 (66, 179)	0–953	800	<0.001
Self-care residents	3 (0, 12)	0–158	22 (4, 53)	0–667	992	<0.001
Partially dependent residents	6 (1, 21)	0–260	35 (18, 68)	0–227	868	<0.001
Dependent residents	11 (1, 24)	0–1,040	46 (13, 76)	0–325	1,048	<0.001
Average monthly fee, all residents (RMB)	4,220 (3,399, 5,066)	1,000–8,400	4,500 (3,800, 5,410)	1,200–11,828	1,097	0.066
Average monthly fee, self-care (RMB)	3,250 (2,600, 3,825)	1,000–6,300	3,475 (2,900, 4,412)	1,800–8,900	846	0.191
Average monthly fee, partially dependent (RMB)	4,000 (3,500, 4,541)	1,200–8,500	4,500 (3,850, 5,294)	2,600–11,000	859	0.012
Average monthly fee, dependent (RMB)	5,000 (4,012, 5,588)	1,838–10,300	5,350 (4,650, 6,000)	3,400–14,213	879	0.018
Medical doctors	19 (8, 38)	0–377	2 (0, 11)	0–23	3,218	<0.001
Medical nurses	21 (12, 50)	0–419	4 (0, 22)	0–59	2,969	<0.001
Medical technicians	5 (2, 13)	0–129	1 (0, 6)	0–25	2,772	<0.001
Medical care assistants	0 (0, 6)	0–315	0 (0, 10)	0–97	1,904	0.912
Elderly-care doctors	1 (0, 2)	0–25	0 (0, 1)	0–12	2,402	0.013
Elderly-care nurses	2 (0, 5)	0–19	2 (0, 6)	0–21	1,859	0.740
Elderly-care technicians	0 (0, 1)	0–16	0 (0, 0)	0–7	2,030	0.509
Elderly-care attendants	5 (2, 10)	0–36	17 (12, 38)	0–111	718	<0.001

Analytic sample sizes were 77 medical-run care and 50 care-run medical institutions unless otherwise specified. Medical-bed counts were available for 76 and 50 institutions, respectively. Monthly-fee comparisons included only positive recorded fee values; the corresponding sample sizes were 59 and 47 for all residents, 48 and 42 for self-care residents, 53 and 46 for partially dependent residents, and 54 and 45 for dependent residents. Zero and missing fee values were excluded from these comparisons.

**Table 2b:** Size-standardised bed and staffing indicators by institutional model.

Variable	Medical-run care		Care-run medical		Mann–Whitney *U* tests	*P* value
	*n*	P50 (P25, P75)	*n*	P50 (P25, P75)		
Medical beds, % of total beds	76	62.50 (50.72, 75.28)	50	12.90 (0.00, 34.74)	3,394	<0.001
Long-term-care beds, % of total beds	77	37.50 (25.00, 50.00)	50	87.10 (65.26, 100.00)	445.5	<0.001
Medical doctors per 100 beds	77	13.56 (7.89, 23.17)	50	1.12 (0.00, 2.84)	3,624	<0.001
Medical nurses per 100 beds	77	18.79 (10.00, 27.38)	50	2.22 (0.00, 7.73)	3,393.5	<0.001
Medical technicians per 100 beds	77	4.00 (2.14, 8.53)	50	0.42 (0.00, 1.39)	3,123	<0.001
Total medical staff per 100 beds†	77	39.19 (21.88, 59.38)	50	4.97 (0.00, 12.04)	3,530.5	<0.001
Elderly-care attendants per 100 beds	77	3.05 (0.94, 6.67)	50	9.40 (5.71, 13.96)	888	<0.001
Elderly-care attendants per 100 residents	64	19.59 (10.67, 30.31)	49	19.05 (12.41, 23.53)	1,609	0.814

Values are median (P25, P75). Ratios were calculated at the facility level before group comparison. Medical-bed proportion was available for 76 medical-run care institutions because one institution had missing medical-bed data. Elderly-care attendants per 100 residents were calculated only for institutions with at least one resident (medical-run care, *n* = 64; care-run medical, *n* = 49). †Total medical staff was defined as medical doctors + medical nurses + medical technicians.

Size-standardised institutional indicators and service-portfolio breadth were compared between medical-run care and care-run medical institutions using the Mann–Whitney *U* test. Domain-specific service provision was compared between organisational models using continuity-corrected chi-square tests or Fisher's exact tests where expected cell counts were small. Domain-specific comparisons were treated as exploratory.

For categorical comparisons in Table 3, Yates’ continuity-corrected chi-square test was used for 2 × 2 contingency tables, whereas Pearson's chi-square test was used for tables with more than two categories. Multivariable logistic regression was then used to estimate adjusted odds ratios (aORs) and 95% confidence intervals (CIs) for factors associated with willingness to choose a community home-based IMEC model. The primary adjusted model was specified on conceptual grounds and with reference to prior literature on integrated care utilisation and willingness. It included sex, chronic disease burden, ADL status, monthly out-of-pocket medical expenditure, signed family doctor/community team contract, awareness of community-based integrated care, and perceived need for convenient home/community medical services. For this model, high perceived need was defined as reporting that convenient home/community medical services were “very” or “quite” necessary, whereas all other responses were classified as lower perceived need. This grouping was intended to distinguish respondents expressing a clear positive need from those reporting less definite or low need. To assess whether the primary estimate depended on this dichotomisation, an additional sensitivity analysis entered the original five-level perceived-need response as an ordinal variable, ranging from 1 (“not at all necessary”) to 5 (“very necessary”).

**Table 3:** Characteristics of surveyed older adults by willingness to choose a community-based integrated medical and elderly care model (*N* = 2,525).

Characteristic	Total *n* (%)	Willing *n* (%)	Unwilling *n* (%)	*χ*^2^ value	*P* value
Sex				1.19	0.275
Female	1,444 (57.2)	1,007 (57.9)	437 (55.5)		
Male	1,081 (42.8)	731 (42.1)	350 (44.5)		
Living arrangement				10.82	0.055
With spouse/partner	1,216 (48.2)	863 (49.7)	353 (44.9)		
With spouse/partner and children	857 (33.9)	559 (32.2)	298 (37.9)		
Living alone without a caregiver	228 (9.0)	163 (9.4)	65 (8.3)		
With children	104 (4.1)	67 (3.9)	37 (4.7)		
Living alone with a caregiver (e.g., domestic helper)	84 (3.3)	58 (3.3)	26 (3.3)		
With siblings or other relatives	36 (1.4)	28 (1.6)	8 (1.0)		
Main caregiver for daily life				4.50	0.342
Self-care	1,423 (56.4)	991 (57.0)	432 (54.9)		
Spouse/partner	606 (24.0)	422 (24.3)	184 (23.4)		
Adult children	446 (17.7)	289 (16.6)	157 (19.9)		
Paid caregiver/domestic helper	28 (1.1)	21 (1.2)	7 (0.9)		
Siblings or other relatives	22 (0.9)	15 (0.9)	7 (0.9)		
Number of chronic conditions				0.65	0.723
None	1,046 (41.4)	711 (40.9)	335 (42.6)		
1–2	1,279 (50.7)	887 (51.0)	392 (49.8)		
≥3	200 (7.9)	140 (8.1)	60 (7.6)		
Activities of daily living				4.96	0.084
Fully independent	2,097 (83.0)	1,446 (83.2)	651 (82.7)		
Partially dependent	317 (12.6)	207 (11.9)	110 (14.0)		
Fully dependent	111 (4.4)	85 (4.9)	26 (3.3)		
Disability level				4.56	0.336
No	2,007 (79.5)	1,387 (79.8)	620 (78.8)		
Mild	242 (9.6)	159 (9.1)	83 (10.5)		
Moderate	138 (5.5)	95 (5.5)	43 (5.5)		
Severe	99 (3.9)	74 (4.3)	25 (3.2)		
Unknown	39 (1.5)	23 (1.3)	16 (2.0)		
Monthly out-of-pocket medical expenditure (RMB)				8.19	0.146
<100	835 (33.1)	547 (31.5)	288 (36.6)		
100–499	1,096 (43.4)	766 (44.1)	330 (41.9)		
500–999	346 (13.7)	253 (14.6)	93 (11.8)		
1,000–1,999	163 (6.5)	112 (6.4)	51 (6.5)		
2,000–2,999	51 (2.0)	37 (2.1)	14 (1.8)		
≥3,000	34 (1.3)	23 (1.3)	11 (1.4)		
Signed family doctor/community team contract				6.12	0.013
Yes	1,971 (78.1)	1,381 (79.5)	590 (75.0)		
No	554 (21.9)	357 (20.5)	197 (25.0)		
Awareness of community-based integrated care				27.27	<0.001
Yes	1,641 (65.0)	1,188 (68.4)	453 (57.6)		
No	884 (35.0)	550 (31.6)	334 (42.4)		
Current main care arrangement				26.55	<0.001
Family care at home	1,432 (56.7)	933 (53.7)	499 (63.4)		
Living at home with home-based services	545 (21.6)	405 (23.3)	140 (17.8)		
Community day care	81 (3.2)	68 (3.9)	13 (1.7)		
Institutional care within the community	28 (1.1)	19 (1.1)	9 (1.1)		
Other	439 (17.4)	313 (18.0)	126 (16.0)		
Perceived need for convenient home/community medical services				123.43	<0.001
Very	1,495 (59.2)	1,127 (64.8)	368 (46.8)		
Quite	600 (23.8)	402 (23.1)	198 (25.2)		
Moderately	291 (11.5)	156 (9.0)	135 (17.2)		
Not very necessary	98 (3.9)	38 (2.2)	60 (7.6)		
Not at all	41 (1.6)	15 (0.9)	26 (3.3)		
Acceptable monthly payment for integrated services (RMB)				27.97	<0.001
<500	1,778 (70.4)	1,168 (67.2)	610 (77.5)		
500–999	530 (21.0)	405 (23.3)	125 (15.9)		
1,000–1,999	167 (6.6)	128 (7.4)	39 (5.0)		
2,000–2,999	26 (1.0)	20 (1.2)	6 (0.8)		
≥3,000	24 (1.0)	17 (1.0)	7 (0.9)		
Preferred payment method for integrated services				8.19	0.017
Health insurance payment	2,308 (91.4)	1,605 (92.3)	703 (89.3)		
Out-of-pocket cash payment	118 (4.7)	77 (4.4)	41 (5.2)		
Other	99 (3.9)	56 (3.3)	43 (5.5)		

Data are *n* (%), unless otherwise stated. “Willing” and “Unwilling” refer to whether respondents reported being willing to choose a community-based integrated medical and care model. *P* values are from Yates’ continuity-corrected chi-square tests for 2 × 2 tables and Pearson's chi-square tests for contingency tables with more than two categories.

Variables such as living arrangement, main caregiver, current care arrangement, acceptable payment level, and preferred payment method were retained in descriptive and univariable analyses but were not simultaneously entered into the primary adjusted model because several were conceptually close to willingness itself or reflected downstream care preferences, and inclusion of all such variables in a single cross-sectional model would have reduced parsimony and interpretability.

Missing data were assessed before modelling. No missing values were observed for variables retained in the final adjusted model, and the complete-case model therefore included all 2,525 respondents. For multi-level categorical predictors, the overall contribution of each factor was assessed using likelihood-ratio tests. Multicollinearity was assessed using variance inflation factors (VIFs), model goodness of fit was examined using the Hosmer–Lemeshow goodness-of-fit test, and model discrimination was evaluated using the area under the receiver operating characteristic curve. Because perceived need for convenient home/community medical services was conceptually close to willingness to choose community-based IMEC, we conducted a sensitivity analysis excluding this variable from the multivariable logistic regression model. This sensitivity model used the same covariates as the primary adjusted model except for perceived need. Community identifiers were not retained in the final analytic dataset; therefore, within-community clustering could not be accounted for using cluster-robust standard errors, GEE or multilevel modelling. Statistical significance was defined as a two-sided *p* value <0.05. All analyses were conducted using SPSS version 26.0.

### 2.6 Quality control

All investigators received standardised training before formal data collection. The questionnaires were pre-tested before field implementation and revised where necessary. During data collection, completed questionnaires were checked for completeness and internal consistency. After fieldwork, data were entered with consistency checking, and selected records were rechecked against the original questionnaires to improve data accuracy.

### 2.7 Ethics

The study was approved by the Ethics Committee of Sichuan Provincial Women's and Children's Hospital (approval no. 20230331-047). The study was conducted in accordance with the local legislation and institutional requirements. Written informed consent was obtained from all participants before data collection. Organisational consent was obtained from participating institutions before the facility survey. All data were anonymised before analysis, and confidentiality was maintained throughout the study.

## 3 Results

The results are presented in four parts: institutional characteristics, resident characteristics by willingness, parallel service-domain patterns, and multivariable analysis of willingness.

### 3.1 Institutional characteristics by organisational model

All 132 registered IMEC institutions were approached. Of these, 127 returned questionnaires with sufficient information for analysis and were included in the analytic sample, including 77 medical-run care institutions and 50 care-run medical institutions; five did not return questionnaires with sufficient information for analysis. Clear structural differences were observed between the two organisational models (Tables 2a, b). Compared with medical-run care institutions, care-run medical institutions had larger overall capacity, with higher median numbers of total beds (206 vs. 150, *p* = 0.032), long-term care beds (155 vs. 32, *p* < 0.001) and total residents (122 vs. 30, *p* < 0.001). They also accommodated more residents at all functional levels, including self-care residents (22 vs. 3, *p* < 0.001), partially dependent residents (35 vs. 6, *p* < 0.001) and dependent residents (46 vs. 11, *p* < 0.001).

Size-standardised analyses confirmed a substantially more medically oriented bed and staffing profile among medical-run care institutions. Medical beds accounted for a median of 62.50% (IQR: 50.72–75.28) of total beds in medical-run care institutions compared with 12.90% (0–34.74) in care-run medical institutions (*p* < 0.001). Conversely, long-term-care beds accounted for 37.50% (25.00–50.00) and 87.10% (65.26–100.00), respectively (*p* < 0.001). Medical-run care institutions also had substantially higher densities of medical doctors (13.56 vs. 1.12), nurses (18.79 vs. 2.22), technicians (4.00 vs. 0.42), and combined medical staff (39.19 vs. 4.97) per 100 beds (all *p* < 0.001). Care-run medical institutions had a higher density of elderly-care attendants relative to total bed capacity (9.40 vs. 3.05 per 100 beds, *p* < 0.001). However, attendant density relative to actual residents did not differ significantly between the two models (19.05 vs. 19.59 per 100 residents, *p* = 0.814). Thus, the higher absolute number of attendants in care-run medical institutions appears to reflect, at least partly, their larger long-term-care capacity rather than greater attendant staffing intensity per resident.

Among institutions with positive recorded fee values, monthly fees were broadly similar between the two models overall, but care-run medical institutions charged higher fees for residents with greater dependency. Median monthly fees for partially dependent residents were RMB 4,500 in care-run medical institutions vs. RMB 4,000 in medical-run care institutions (*p* = 0.012), and for dependent residents RMB 5,350 vs. RMB 5,000 (*p* = 0.018). By contrast, average monthly fees for all residents and for self-care residents did not differ significantly between the two organisational models. Overall, these findings suggest that institutions operating under the same IMEC label differed substantially in scale, staffing configuration and functional orientation.

Service-portfolio breadth was broadly similar between the two organisational models. Medical-run care institutions reported a median of 11.0 (IQR: 8.0–12.0) of 12 core service domains considered needed by community-dwelling older adults, compared with 10.5 (8.25–12.0) among care-run medical institutions (*p* = 0.365). The corresponding numbers of service domains currently able to be provided were 4.0 (0–8.0) and 5.0 (0–8.75), respectively (*p* = 0.791). Domain-specific comparisons are presented in Supplementary Table S2. Overall, these analyses did not indicate a consistent difference in service-portfolio breadth between the two organisational models.

### 3.2 Characteristics of surveyed older adults by willingness to choose community-based IMEC

Among the 2,525 surveyed older adults, 1,738 (68.8%) reported that they would be willing to choose a community-based integrated medical and elderly care model, while 787 (31.2%) reported unwillingness (Table 3). Most socio-demographic and health-related characteristics were not significantly associated with willingness. There were no statistically significant differences between willing and unwilling respondents in sex (*p* = 0.275), living arrangement (*p* = 0.055), main caregiver for daily life (*p* = 0.342), number of chronic conditions (*p* = 0.723), disability level (*p* = 0.336) or monthly out-of-pocket medical expenditure (*p* = 0.146). Functional dependence in ADL showed only a borderline association (*p* = 0.084).

In contrast, several service-related and contextual factors differed significantly between the two groups. Respondents with a signed family doctor/community team contract were more likely to report willingness than those without such a contract (70.1% vs. 64.4%, *p* = 0.013). Awareness of community-based integrated care also differed markedly: 68.4% of willing respondents were aware of this model, compared with 57.6% among unwilling respondents (*p* < 0.001). Current main care arrangement was strongly associated with willingness (*p* < 0.001); compared with unwilling respondents, willing respondents were less likely to rely exclusively on family care at home (53.7% vs. 63.4%) and more likely to report receiving home-based services provided by community staff or local care institutions (23.3% vs. 17.8%) or using community day care (3.9% vs. 1.7%).

Perceived need for convenient home/community medical services showed the strongest univariable association with willingness (*p* < 0.001). Among willing respondents, 64.8% rated such services as “very” necessary, compared with 46.8% among unwilling respondents, whereas unwilling respondents were more likely to report lower levels of need. Financial expectations also differed between groups. Willing respondents were more likely to accept a monthly payment of RMB 500–999 for integrated services (23.3% vs. 15.9%), while unwilling respondents were more concentrated in the “<500 RMB” category (77.5% vs. 67.2%) (*p* < 0.001). Preferred payment method also differed significantly (*p* = 0.017), with willing respondents more likely to favour health insurance payment (92.3% vs. 89.3%). Taken together, these results suggest that willingness to choose community-based IMEC is more closely associated with awareness, perceived relevance and service/payment expectations than with the measured demographic or clinical characteristics.

### 3.3 Parallel service-domain patterns in institutional capacity and resident-reported receipt and importance

Table 4 presents institutional and resident service-domain indicators in parallel. These indicators represent different constructs and should not be interpreted as directly comparable measures of supply and demand. In particular, resident-reported receipt was not linked to the surveyed IMEC institutions and may have reflected services obtained from other providers.

**Table 4:** Parallel descriptive presentation of institutional service capacity and residents’ service receipt and perceived importance across selected care domains [*n* (%)].

Service domain	Institutions considering service needed	Institutions currently able to provide service	Residents ever received service	Residents consider service important
Palliative care	81 (63.8)	17 (13.4)	—	
Health record establishment	92 (72.4)	56 (44.1)	—	
Personal care/ADL support	109 (85.8)	56 (44.1)	334 (13.2)	-
Health assessment	109 (85.8)	61 (48.0)	—	
Medical services	109 (85.8)	55 (43.3)	1,349 (53.4)	1,960 (77.6)
Health examinations	97 (76.4)	52 (40.9)	1,655 (65.5)	1,711 (67.8)
Family beds/home-based medical services	74 (58.3)	12 (9.4)	914 (36.2)	1,659 (65.7)
Rehabilitation technical guidance	106 (83.5)	54 (42.5)	734 (29.1)	1,471 (58.3)
Chronic disease management	115 (90.6)	62 (48.8)	—	
Psychological/spiritual support	110 (86.6)	57 (44.9)	721 (28.6)	971 (38.5)
Nursing	103 (81.1)	54 (42.5)	—	
Health education	106 (83.5)	65 (51.2)	1,104 (43.7)	1,242 (49.2)

The institutional and resident indicators are not directly equivalent. Resident-reported service receipt was not linked to the surveyed IMEC institutions and may have originated from other providers. The table is therefore intended as a parallel descriptive presentation and should not be interpreted as a direct supply–demand comparison. A dash indicates that a directly corresponding resident-side indicator was not collected.

Within the institutional survey, substantial perceived-need–capacity differences were observed across several service domains. For most domains, the proportion of institutions considering a service to be needed by community-dwelling older adults exceeded the proportion reporting that they were currently able to provide it, indicating substantial perceived-need–capacity differences. Using 127 institutions as the denominator for all institutional percentages, these differences were particularly large for palliative care (63.8% vs. 13.4%), family beds/home-based medical services (58.3% vs. 9.4%), medical services (85.8% vs. 43.3%), rehabilitation technical guidance (83.5% vs. 42.5%), psychological/spiritual support (86.6% vs. 44.9%) and health education (83.5% vs. 51.2%). Health record establishment showed a similar perceived-need–capacity difference: 92 institutions (72.4%) considered this service to be needed by community-dwelling older adults, whereas 56 institutions (44.1%) reported being currently able to provide it.

Within the household survey, substantial importance–receipt differences were observed for several community-oriented services. Health examinations and medical services were the most commonly received services and were also among the most highly valued. Specifically, 65.5% of residents had ever received health examinations and 67.8% considered them important, while 53.4% had ever received medical services and 77.6% considered them important. However, several community-oriented and enabling services displayed marked importance–receipt differences. Family beds/home-based medical services were considered important by 65.7% of residents, yet only 36.2% had ever received them. Rehabilitation technical guidance was regarded as important by 58.3% but had been received by only 29.1%. Likewise, psychological/spiritual support showed a difference between perceived importance (38.5%) and actual receipt (28.6%). Health education showed a smaller but still relevant difference (49.2% important vs. 43.7% ever received).

Overall, the two surveys revealed distinct but parallel descriptive patterns. Institutions commonly reported a difference between the services they believed needed and those they were currently able to provide, while residents reported differences between services they considered important and those they had ever received. Because service receipt among residents was not linked to the surveyed institutions, these patterns should not be interpreted as direct evidence of supply–demand mismatch.

### 3.4 Factors associated with willingness to choose community-based IMEC

The final complete-case multivariable model included all 2,525 respondents because no missing values were observed for variables retained in the adjusted model. No evidence of problematic multicollinearity was detected, with VIFs ranging from 1.008 to 1.494. The Hosmer–Lemeshow test did not detect evidence of poor fit (*χ*^2^ = 3.66, df = 8, *p* = 0.886). The area under the receiver operating characteristic curve was 0.627, indicating modest discrimination.

In multivariable logistic regression analyses (Table 1), high perceived need for convenient home/community medical services showed the strongest association with willingness to choose a community-based integrated model (aOR: 2.76, 95% CI: 2.23–3.42, *p* < 0.001). Awareness of community-based integrated care was also associated with higher willingness after adjustment (aOR: 1.55, 95% CI: 1.28–1.87, *p* < 0.001). In addition, respondents with monthly out-of-pocket medical expenditure of RMB 500–999 were more likely to report willingness than those spending <100 RMB per month (aOR: 1.38, 95% CI: 1.01–1.87, *p* = 0.040). By contrast, other expenditure categories were not significantly associated with willingness. However, an omnibus likelihood-ratio test showed that monthly OOP expenditure was not significantly associated with willingness as an overall categorical factor (*χ*^2^ = 4.72, df = 5, *p* = 0.451). The isolated RMB 500–999 category-level association should therefore be interpreted cautiously. Chronic disease burden was also non-significant overall (*χ*^2^ = 0.11, df = 2, *p* = 0.946), while the overall ADL factor did not reach conventional statistical significance (*χ*^2^ = 5.29, df = 2, *p* = 0.071).

Among the measured covariates included in the adjusted model, sex, chronic disease burden, ADL status and family doctor/community team contract status were not significantly associated with willingness. Female sex was not significant (aOR: 1.10, 95% CI: 0.92–1.31, *p* = 0.280), nor were chronic disease burden (1–2 chronic conditions: aOR: 1.03, 95% CI: 0.84–1.25, *p* = 0.796; ≥3 chronic conditions: aOR: 0.98, 95% CI: 0.66–1.45, *p* = 0.928) or ADL dependence (vs. partially dependent: aOR: 0.78, 95% CI: 0.59–1.03, *p* = 0.085; vs. fully dependent: aOR: 1.34, 95% CI: 0.82–2.18, *p* = 0.247). Similarly, signing a family doctor/community team contract did not remain significant in the adjusted model (aOR: 1.01, 95% CI: 0.81–1.26, *p* = 0.895).

In the sensitivity analysis excluding perceived need for convenient home/community medical services (Supplementary Table S3), awareness of community-based integrated care remained significantly associated with willingness (aOR: 1.56, 95% CI: 1.30–1.88, *p* < 0.001). Monthly out-of-pocket medical expenditure of RMB 500–999 also remained associated with willingness compared with <100 RMB per month (aOR: 1.48, 95% CI: 1.09–1.99, *p* = 0.011). Other measured covariates in the sensitivity model remained non-significant. Model discrimination was limited (area under the ROC = 0.582), reinforcing the interpretation of these findings as adjusted associations rather than individual-level predictions. In an additional sensitivity analysis using the original five-level perceived-need response as an ordinal measure (Supplementary Table S4), each one-category increase in perceived need remained associated with higher willingness to choose community-based IMEC (aOR: 1.59, 95% CI: 1.45–1.73, *p* < 0.001), indicating that the association was not dependent solely on the binary cut-point used in the primary model.

Taken together, perceived need and awareness showed the most consistent associations with willingness, whereas the measured health-related covariates and OOP expenditure as an overall factor were not significantly associated with willingness. This pattern suggests that willingness reflects perceived service relevance and service visibility more than conventional indicators of health status alone.

## 4 Discussion

### 4.1 Principal findings

This study provides a dual-perspective assessment of community-based IMEC in Chengdu by analysing institutional and resident survey data in parallel. Three principal findings emerged. First, willingness to choose community-based IMEC was relatively high, with 68.8 per cent of surveyed older adults reporting willingness to use this model. Second, willingness was associated more strongly with awareness of community-based IMEC and perceived need for convenient home- and community-based medical services than with the measured health-related covariates included in the model. Third, substantial organisational heterogeneity was observed, while the two surveys separately identified perceived-need–capacity differences within institutions and importance–receipt differences among residents. These patterns were particularly apparent in several home- and community-oriented service domains relevant to ageing in place and care continuity. Taken together, these findings suggest that the central challenge is not simply whether older adults are open to integrated care, but whether existing community-based IMEC arrangements have the organisational, service and information-related capacities needed to support continuous and community-oriented care.

### 4.2 Interpreting willingness: associations with perceived need and awareness

Perceived need and awareness were associated with willingness in the adjusted model, whereas the measured health-related covariates did not show statistically significant associations. These findings highlight perceived need and awareness as potential correlates of willingness, without establishing that they are more important than clinical or sociodemographic characteristics. This interpretation is consistent with broader integrated care literature, which has emphasised that service uptake depends not only on formal availability, but also on perceived usefulness, accessibility and person-centred relevance (6, 7).

This finding is also broadly consistent with recent evidence from China. Studies of home- and community-based medical and elderly care services have shown that utilisation is often more closely related to service accessibility, information and perceived usefulness than to clinical need alone (32–34). Likewise, discrete choice evidence suggests that older and middle-aged adults in China make explicit trade-offs between service attributes such as medical capacity, service quality and cost when evaluating IMEC institutions (35). Our results extend this literature by showing that, even in a large urban setting with active policy development, willingness remains strongly conditioned by whether the service model is known and perceived as practically useful.

At the same time, this finding should be interpreted cautiously. Perceived need for convenient home/community medical services is conceptually close to willingness to choose community-based IMEC, and the strong observed association may therefore partly reflect overlap between perceived service relevance and stated willingness rather than a wholly independent explanatory mechanism. To examine this issue, we conducted a sensitivity analysis excluding perceived need from the adjusted model. In this sensitivity model, awareness of community-based integrated care remained significantly associated with willingness. Although the RMB 500–999 OOP category retained a category-specific association, OOP expenditure was not significant as an overall factor and was therefore not interpreted as a principal correlate of willingness. These findings suggest that awareness and service visibility were not solely artefacts of the perceived-need variable. However, the reduced model showed limited discrimination, and the regression findings should therefore be interpreted as adjusted associations rather than as evidence of causal determinants or individual-level prediction.

### 4.3 Organisational heterogeneity and the limits of integration “in name”

The comparison between medical-run care institutions and care-run medical institutions highlights a second important issue: The size-standardised analyses reinforce the distinction in bed and medical staffing structure between the two organisational models, but also refine the interpretation of their care orientation. Medical-run care institutions retained markedly higher medical-bed proportions and medical staffing densities after accounting for institutional size, supporting a more medically oriented organisational profile. Care-run medical institutions allocated a substantially larger share of their beds to long-term care and had more elderly-care attendants relative to bed capacity; however, attendant density per resident did not differ between the two models. The distinction therefore appears to lie more clearly in bed allocation and medical staffing configuration than in the intensity of direct-care staffing per resident. Despite these structural differences, overall service-portfolio breadth did not differ significantly between the two organisational models. This suggests that institutional origin was more clearly associated with bed composition and staffing configuration than with the overall number of service domains that institutions considered needed by community-dwelling older adults or were currently able to provide (27, 31, 36).

This heterogeneity is not merely descriptive; it has direct implications for implementation. If organisational models differ substantially in workforce composition and bed structure, they are unlikely to deliver equivalent packages of community-oriented support. In this sense, the policy label of IMEC may conceal considerable variation in actual service capacity. This interpretation is consistent with previous analyses showing that integrated medical and elderly care in China has progressed unevenly across institutional types and localities, with continuing constraints related to workforce shortages, financing arrangements and unclear divisions of sectoral responsibility (22, 27, 37). International experience also suggests that integrated care reforms do not produce uniform benefits across settings; rather, their effects are often mixed and shaped by local financing, service organisation, and implementation capacity (14–16). Our findings therefore suggest that “integration in name” does not necessarily translate into integration in practice, and that this challenge is not unique to the Chinese setting.

### 4.4 Parallel service-capacity and service-receipt patterns relevant to ageing in place

A third contribution of this study is the parallel description of service-related patterns from institutional and resident perspectives. Within institutions, the reported ability to provide several services was substantially lower than the proportion of institutions that considered those services to be needed by community-dwelling older adults. Separately, residents reported marked differences between the services they considered important and those they had ever received. These two patterns should not be interpreted as direct counterparts, because resident-reported receipt was not linked to the surveyed institutions. At the resident level, marked importance–receipt differences were observed for home-based medical services, rehabilitation technical guidance, psychological/spiritual support and health education. These are not peripheral services. They are central to enabling older adults with chronic conditions or emerging functional limitations to remain safely and meaningfully in their homes and communities.

This pattern matters because it suggests that current community-based IMEC provision may remain biased towards conventional medical and examination-oriented services, while lagging behind in the more enabling, continuity-oriented and supportive functions that are essential to ageing in place. International integrated care frameworks have repeatedly emphasised the importance of coordinated home-based support, rehabilitation, psychosocial care and functional maintenance for older populations (7, 11, 12). Our results indicate that these functions remain incompletely institutionalised in the Chengdu setting, despite broad recognition of their importance.

The findings also resonate with recent Chinese evidence showing unmet community-care needs among older adults and differences between older adults’ care needs and available community services (33, 38). In practice, such differences may reflect several intersecting constraints: insufficient workforce with community rehabilitation or psychosocial skills, limited incentives for institutions to provide low-margin home-based services, and weak service navigation mechanisms that connect households to appropriate support. As a result, service packages may remain more institution-centred than community-oriented, even under a policy framework explicitly designed to promote integration.

### 4.5 Data-supported continuity of care and implications for personal health data spaces

The findings also have conceptual implications for data-supported continuity of care. In the institutional survey, 72.4% of institutions reported that health record establishment was needed by community-dwelling older adults, whereas 44.1% reported being currently able to provide this service. This identifies a difference between institution-perceived resident need and reported capacity for a basic record-establishment function. However, the survey did not establish the type, quality, updating practices or exchangeability of these records.

From the perspective of PHDS-enabled care continuity, this finding is important because personal health data spaces require more than technical platforms alone; they also depend on interoperability, data governance, privacy protection and organisational routines that allow usable health information to support care coordination across settings (18–20). They also depend on service organisations that can generate reliable health and care information, use it in routine practice and coordinate care across providers and settings. In the context of older adults with chronic conditions, functional decline or long-term support needs, weak health-record capacity may limit the ability of community-based IMEC institutions to support continuity between home-based care, community services, outpatient care and institutional support. Accordingly, this finding should not be interpreted as evidence of PHDS readiness or electronic health-record interoperability. Rather, it identifies a basic information-recording function that may form part of the broader organisational foundation required for future data-supported continuity of care.

However, the present study did not directly evaluate PHDS implementation. We did not measure electronic health record interoperability, individual access to or control over personal health data, consent architecture, privacy governance or real-time data exchange between providers. The contribution of this study is therefore more limited and health-services oriented: it identifies organisational and service-capacity differences, including limited health record establishment, that are relevant to the future development of data-supported continuity of care in community-based IMEC.

### 4.6 Policy implications

These findings have several implications for policy and service design. First, they suggest that reform should move beyond expanding the number of IMEC institutions and focus more directly on the content of the service package delivered at community level. If community-based IMEC is intended to support ageing in place, then home-based medical care, rehabilitation support, chronic disease management, psychosocial care and caregiver-oriented support should be treated as core functions rather than optional extensions.

Second, the strong association between awareness and willingness indicates that information environments matter. Community-based IMEC cannot be effective if older adults and families do not understand what the model offers, who it is intended for or how services can be accessed. This suggests a role for family doctor teams, community health workers, hospital discharge planning and neighbourhood-level service navigation in making integrated care more visible and intelligible. However, the present study cannot demonstrate that increasing awareness would directly increase subsequent utilisation; this should be understood as a policy-relevant hypothesis rather than a causal inference from the current data. In the longer term, these navigation functions could be strengthened by data-supported care coordination, including more systematic health record establishment and appropriate information-sharing mechanisms between community IMEC institutions, primary care teams and other health or long-term care providers.

Third, the observed organisational heterogeneity implies a need for clearer service standards and stronger alignment between regulatory approval and functional expectations. Institutions with different historical origins may require differentiated staffing requirements, supervision standards and purchasing arrangements if they are to deliver comparable community-oriented services. Payment reform may be particularly important. Recent evidence on long-term care insurance in China indicates that financing arrangements can influence healthcare utilisation and patterns of formal care use among older adults (29, 30). Strengthening links between IMEC, health insurance and longer-term care financing could therefore help reduce access barriers and support more sustainable service delivery.

Finally, Chengdu's marked intra-city variation in the proportion of adults aged 65 years and older suggests that community-based IMEC planning should also be spatially differentiated. Outer-ring districts and county-level cities have older age structures than many inner-ring areas, which implies potentially greater demand for community-oriented long-term support and home-based services. A more place-sensitive allocation of institutions, workforce and outreach capacity may therefore be needed to avoid reproducing geographical inequalities in integrated care access.

### 4.7 Strengths and limitations

This study has several strengths. It uses two parallel surveys within the same urban setting to characterise institutional configurations and older adults’ willingness and service experiences. It also includes a comparatively large sample of institutions and older adults, allowing organisational heterogeneity and resident-level willingness to be examined within a common health-services context.

Several limitations should be acknowledged. First, the cross-sectional design precludes causal inference; associations between awareness, perceived need and willingness should not be interpreted as causal effects. Second, perceived need for convenient home/community medical services is conceptually close to willingness itself, and the observed association may partly reflect overlap between the constructs. Although the adjusted model showed acceptable calibration and no evidence of problematic multicollinearity, its discrimination was modest, and the sensitivity model excluding perceived need also had limited discrimination. The regression results should therefore be interpreted as exploratory adjusted associations rather than as causal determinants or an individual-level prediction tool. Third, the study was conducted in one city, and the findings may not be generalisable to areas with different demographic structures, service systems or policy histories. The resident sample was geographically distributed across Chengdu but was recruited using a convenience-based approach rather than a random, quota-based or population-weighted probability design. Counts of older adults approached, refusals, non-response and non-participation were not retained; representativeness and selection bias should therefore be interpreted cautiously. In addition, potential within-community clustering could not be accounted for because community identifiers were not retained; this may have resulted in overly precise standard errors and confidence intervals in the regression analyses. Moreover, exact age, education, household income and geographic residence were not collected, preventing adjustment for these potentially important sociodemographic and geographic confounders and leaving the regression estimates susceptible to residual confounding. Fourth, the institutional and resident surveys were not linked at the district, community or facility-catchment level, so the service-comparison analysis is descriptive and cannot demonstrate that specific local institutional configurations directly produced individual-level willingness or utilisation patterns. Fifth, key variables, including awareness, perceived need and willingness, were self-reported and may be affected by reporting or social desirability bias. Sixth, willingness to choose community-based IMEC does not necessarily translate into actual utilisation, especially where service availability, family decision-making or affordability constrain behaviour. Future studies would benefit from longitudinal designs, direct geographic linkage between residents and nearby IMEC facilities, district-level policy and financing measures, and closer linkage between stated preferences and actual service use. Seventh, although we discuss implications for data-supported continuity of care and PHDS development, the study did not directly measure electronic health record interoperability, individual access to or control over health data, consent and privacy mechanisms, or cross-provider data exchange. In addition, the health-record-establishment item did not distinguish paper-based from electronic records or assess record-updating practices. The connection to PHDSs should therefore be interpreted as conceptual and service-system oriented rather than as direct empirical evidence on PHDS implementation.

## 5 Conclusion

In conclusion, community-based IMEC in Chengdu shows substantial latent acceptability among older adults, together with marked organisational heterogeneity. Within institutions, important perceived-need-capacity differences were observed across several service domains, while residents separately reported importance–receipt differences for a range of community-oriented services. Because resident-reported service receipt was not linked to the surveyed institutions, these findings should be interpreted as parallel descriptive patterns rather than as evidence of direct supply–demand mismatch. For community-based IMEC to support continuity of care more effectively, future reform should focus on strengthening community-oriented service packages, improving service visibility and navigation, and building the organisational and information-related capacities needed for data-supported coordination across home, community, outpatient and institutional care settings.

## Data Availability

The datasets presented in this article are not readily available because they contain potentially identifiable information on older adults and participating institutions and are subject to ethics and administrative restrictions. Requests to access the datasets should be directed to Tongyong Luo (whnic@msn.com). Access may be granted subject to institutional approval and applicable ethics requirements.
