# Supplementary material for: Community-based integrated medical and elderly care in Chengdu, China: organisational heterogeneity, service-capacity and service-receipt patterns, and implications for care continuity
Source: Front Health Serv. 2026 Oct 19;6:1875860. doi: 10.3389/frhs.2026.1875860 (PMC13724404; doi:10.3389/frhs.2026.1875860)
Supplement: Supplementary file 1 [file Table1.docx]

**Supplementary Table S1. Operational definitions and coding of key institutional and resident survey measures**

**Panel A. Institutional service-domain measures**

| **Measure / service domain** | **Original survey construct** | **Response options and coding** | **Analytic interpretation** |
| --- | --- | --- | --- |
| Institution-perceived resident need for each service domain | For each listed domain, the institution indicated whether it considered the service to be needed by community-dwelling older adults. | Yes = 1; No = 0. | Binary institution-level indicator of perceived resident need. It is not an indicator that the institution itself intended or was normatively required to provide the service. |
| Current ability to provide each service domain | For the corresponding domains, the institution indicated whether it was currently able to provide the community-based service. | Yes = 1; No = 0. | Binary institution-level indicator of current reported service capacity. |
| Service domains assessed | Medical services; psychological/spiritual support; rehabilitation technical guidance; health assessment; nursing services; health education and guidance; health examinations; health record establishment; chronic disease management; family beds; personal care; palliative care; other services. | Each domain recorded separately as yes/no for both institutional indicators. | The same 12 prespecified core domains were used for service-portfolio breadth; 'other services' was retained descriptively and was not included in the 12-domain breadth score. |
| Health record establishment | Establishment of health records was one of the listed institutional service domains. | Yes = 1; No = 0, separately for perceived resident need and current ability to provide. | A basic record-establishment service indicator only. The item did not distinguish paper from electronic records and did not assess record updating, individual access/control, interoperability, consent/privacy mechanisms, or cross-provider data exchange. |

**Panel B. Key resident survey measures**

| **Measure / service domain** | **Original survey construct** | **Response options and coding** | **Analytic interpretation** |
| --- | --- | --- | --- |
| Awareness of community-based integrated care | Question 16: "Had you previously heard of/known about community home-based integrated medical and elderly care services?" | Aware ("了解") = 1; Not aware ("不了解") = 0. | Binary resident-level awareness indicator. |
| Perceived need for convenient home/community medical services | Question 18: "Do you think older adults need convenient medical services that can be accessed at home or within the community?" | Very necessary; Quite necessary; Moderately necessary; Not very necessary; Not at all necessary. Primary model: Very/Quite = high perceived need; all other responses = lower perceived need. Ordinal sensitivity analysis: 1 = not at all necessary to 5 = very necessary. | Resident-level perceived need. The ordinal specification was used in Supplementary Table S4 to assess sensitivity to the primary binary cut-point. |
| Ever received service | Question 19: "Which of the following services have you previously received in your street/community?" (multiple response). | Each selected service = 1; not selected = 0. | Resident-reported prior receipt within the street/community. The questionnaire did not identify the specific provider, so receipt cannot be attributed to the 127 surveyed IMEC institutions. |
| Consider service important | Question 22: "Which of the following community home-based medical services do you consider most important for older adults?" (multiple response). | Each selected service = 1; not selected = 0. | Resident-reported perceived importance of the listed service. |
| Willingness to choose community home-based IMEC | Question 20: "Would you be willing to choose a community home-based integrated medical and elderly care model?" | Yes = 1; No = 0. | Primary binary outcome in the logistic regression. |

**Panel C. Mapping of resident service-response items to analytic service domains**

| **Analytic service domain** | **Resident 'ever received' item (Question 19)** | **Resident 'consider important' item (Question 22)** |
| --- | --- | --- |
| Medical services | Medical diagnosis/treatment (医疗诊治) | Medical diagnosis/treatment (医疗诊治) |
| Health examinations | Health examination (健康体检) | Physical examination (体检) |
| Family beds / home-based medical services | Home-based medical services, including home visits/family beds (上门提供医疗服务〔医生上门巡诊、家庭病床等〕) | Home-based medical services (上门提供医疗服务) |
| Rehabilitation technical guidance | Rehabilitation care (康复护理) | Rehabilitation care (康复护理) |
| Psychological/spiritual support | Psychological counselling / spiritual comfort (心理咨询和/或精神慰藉)* | Psychological counselling / spiritual comfort (心理咨询和/或精神慰藉)* |
| Personal care / ADL support | Personal care (生活照料) | Not included as a separate option in Question 22 |
| Health education | Health consultation and health-preservation lectures (健康咨询与养生讲座) | Health consultation and health-preservation lectures (健康咨询与养生讲座) |

**Notes:** IMEC, integrated medical and elderly care; ADL, activities of daily living. English wording is a faithful translation of the Chinese questionnaire fields available in the analytic datasets. For the institutional dataset, the source spreadsheet explicitly identifies the first set of service-domain variables as services that institutions considered needed by community-dwelling older adults and the second set as community-based extended services that institutions were able to provide. For resident multiple-response questions, non-selection of an option was coded as 0 for that item.
