# Supplementary material for: Community-based integrated medical and elderly care in Chengdu, China: organisational heterogeneity, service-capacity and service-receipt patterns, and implications for care continuity
Source: Front Health Serv. 2026 Oct 19;6:1875860. doi: 10.3389/frhs.2026.1875860 (PMC13724404; doi:10.3389/frhs.2026.1875860)
Supplement: Supplementary file 2 [file Table2.docx]

**Supplementary Table S2. Service-portfolio comparisons between medical-run care and care-run medical institutions**

**Panel A. Overall service-portfolio breadth**

| Variable | Medical-run care, | | Care-run medical | | Mann–Whitney U | P value |
| --- | --- | --- | --- | --- | --- | --- |
|  | n | Median (P25–P75) | n | Median (P25–P75) |  |  |
| Number of service domains considered needed (0–12) | 77 | 11.0 (8.0–12.0) | 50 | 10.5 (8.25–12.0) | 2103 | 0.365 |
| Number of service domains currently able to provide (0–12) | 77 | 4.0 (0–8.0) | 50 | 5.0 (0–8.75) | 1871.5 | 0.791 |

**Panel B. Domain-specific service portfolio comparisons**

| Service domain | Institutions believing service should be needed | | | Institutions currently able to provide service | | |
| --- | --- | --- | --- | --- | --- | --- |
|  | Medical-run care | Care-run medical | P value | Medical-run care | Care-run medical | P value |
|  | (n=77), n (%) | (n=50), n (%) |  | (n=77), n (%) | (n=50), n (%) |  |
| Medical services | 68 (88.3) | 41 (82.0) | 0.462 | 35 (45.5) | 20 (40.0) | 0.672 |
| Psychological/spiritual support | 66 (85.7) | 44 (88.0) | 0.918 | 30 (39.0) | 27 (54.0) | 0.138 |
| Rehabilitation technical guidance | 65 (84.4) | 41 (82.0) | 0.91 | 36 (46.8) | 18 (36.0) | 0.311 |
| Health assessment | 64 (83.1) | 45 (90.0) | 0.409 | 36 (46.8) | 25 (50.0) | 0.86 |
| Nursing | 62 (80.5) | 41 (82.0) | 1 | 32 (41.6) | 22 (44.0) | 0.93 |
| Health education | 64 (83.1) | 42 (84.0) | 1 | 39 (50.6) | 26 (52.0) | 1 |
| Health examinations | 60 (77.9) | 37 (74.0) | 0.768 | 36 (46.8) | 16 (32.0) | 0.142 |
| Health record establishment | 52 (67.5) | 40 (80.0) | 0.183 | 33 (42.9) | 23 (46.0) | 0.868 |
| Chronic disease management | 69 (89.6) | 46 (92.0) | 0.763† | 40 (51.9) | 22 (44.0) | 0.488 |
| Family beds/home-based medical services | 44 (57.1) | 30 (60.0) | 0.893 | 6 (7.8) | 6 (12.0) | 0.538† |
| Personal care/ADL support | 63 (81.8) | 46 (92.0) | 0.178 | 28 (36.4) | 28 (56.0) | 0.046 |
| Palliative care | 55 (71.4) | 26 (52.0) | 0.042 | 9 (11.7) | 8 (16.0) | 0.667 |

**Notes:** Panel A values are median (P25–P75) and were compared using the Mann–Whitney U test. For Panel B, percentages use 77 medical-run care institutions and 50 care-run medical institutions as denominators. Yates continuity-corrected chi-square tests were used for 2×2 comparisons unless an expected cell count was <5, in which case Fisher's exact test was used (†). Domain-specific comparisons were exploratory and were not adjusted for multiple comparisons. The two institutional questionnaire indicators were whether the institution considered a service to be needed by community-dwelling older adults and whether the institution was currently able to provide the corresponding service.
