# Supplementary material for: Community-based integrated medical and elderly care in Chengdu, China: organisational heterogeneity, service-capacity and service-receipt patterns, and implications for care continuity
Source: Front Health Serv. 2026 Oct 19;6:1875860. doi: 10.3389/frhs.2026.1875860 (PMC13724404; doi:10.3389/frhs.2026.1875860)
Supplement: Supplementary file 3 [file Table3.docx]

**Supplementary Table S3.**

**Panel A. Sensitivity analysis excluding perceived need for convenient home/community medical services from the multivariable logistic regression model**

| **Variable** | **aOR** | **95% CI** | **p-value** |
| --- | --- | --- | --- |
| Female vs male | 1.12 | 0.94–1.32 | 0.214 |
| 1–2 chronic conditions vs none | 1.02 | 0.84–1.25 | 0.821 |
| ≥3 chronic conditions vs none | 1.02 | 0.70–1.50 | 0.917 |
| Partially dependent in ADL vs fully independent | 0.78 | 0.59–1.02 | 0.073 |
| Fully dependent in ADL vs fully independent | 1.32 | 0.82–2.14 | 0.258 |
| OOP 100–499 RMB vs <100 | 1.22 | 0.99–1.50 | 0.060 |
| OOP 500–999 RMB vs <100 | 1.48 | 1.09–1.99 | 0.011 |
| OOP 1000–1999 RMB vs <100 | 1.20 | 0.81–1.77 | 0.364 |
| OOP 2000–2999 RMB vs <100 | 1.36 | 0.70–2.62 | 0.364 |
| OOP ≥3000 RMB vs <100 | 1.03 | 0.48–2.19 | 0.941 |
| Signed family doctor/community contract vs no | 1.09 | 0.88–1.35 | 0.434 |
| Aware of community-based integrated care vs not aware | 1.56 | 1.30–1.88 | <0.001 |

**Panel B. Sensitivity model diagnostics**

| Indicator | **Result** |
| --- | --- |
| Complete-case N | 2,525 |
| VIF range | 1.008–1.489 |
| Hosmer–Lemeshow test | χ² = 5.08, df = 8, p = 0.749 |
| AUC | 0.582 |
| McFadden pseudo-R² | 0.014 |
